# New single-switch buck–boost converter with continuous input/output currents and a wide conversion range

**DOI:** 10.1016/j.heliyon.2024.e37236

**Published:** 2024-08-30

**Authors:** Mohammad Lotfi-nejad, Hossein Hajisadeghian, Amir Abbas Aghajani, Ali A. Motie Birjandi, Ehsan Adib

**Affiliations:** a Faculty of Electrical Engineering, Shahid Rajaee Teacher Training University, Tehran, Iran; b Faculty of Electrical Engineering, Isfahan University of Technology, Isfahan, Iran

**Keywords:** Buck-boost, DC-DC converter, Wide conversion ratio, High step-up, Single-switch

## Abstract

The main aim of this paper is to introduce a new high step-up/down buck-boost converter with a minimum number of switches which provides the benefit of having the continuity of the input/output current. This single-switch semi-quadratic converter is suitable for high step-up applications while being able to provide step-down voltage gains. Also, by applying some minor changes to the circuit elements, another single-switch buck-boost converter is suggested which has two operative outputs with different voltage gains. One of the outputs of this topology has quadratic buck-boost converter voltage gain which is appropriate for high step-up/step-down applications. The other output could vary from input voltage to minus infinity, ideally. After studying the steady-state operation of the proposed converters in Continuous Conduction Mode (C.C.M), the simulation results are presented. In addition, a comparison among related converters proposed in the literature is made. Finally, experimental results are evaluated by implementing a laboratory prototype of the proposed converter in both step-down and step-up modes. Theoretical, simulation, and experimental results are compatible with each other.

## 1 Introduction

Nowadays, DC/DC converters are employed in many applications such as electric vehicles, battery chargers, energy storage systems, and microgrids [[1], [2], [3], [4]]. Meanwhile, DC/DC converter topologies with the ability to possess high voltage gains are being developed to lower the demand for cascading converters, and consequently, lowering costs and increase efficiency. As an example, in renewable Photovoltaic (PV) solar energy, the produced voltage from PV arrays needs to be highly stepped up in order to be used [5].In Ref. [6], a novel single switch high step-up boost converter for renewable energy applications is presented. Although a high step-up boost converter is able to achieve a high output voltage level without severe duty cycle problems, they are unsuitable for step-down/step-up applications. Many DC/DC converter applications require a wide range of regulated output voltage (or current) within a wide range of input voltages. Some examples of this application are renewable energies, uninterruptible power supplies, railways, electric cars, industrial devices, and near-space vehicle systems in which, as a solution, different buck-boost topologies have been proposed [[7], [8], [9], [10], [11]]. While studying the proposed topologies of buck-boost type converters in literature, different aspects like the number of power electronic switches, number of diodes, static voltage gain, continuity of input and output current, and output polarity have been taken into account. The majority of studies focused on buck-boost converters with high step-up gain while using the minimum power electronic switches. Higher step-up (or higher step-down) gains assist converters in achieving more comprehensive output voltage ranges without extreme duty cycles [[12], [13], [14], [15]]. Also, using the minimum power electronic switches presents a more straightforward and lower-cost controller for converters [16]. [17] presents a non-isolated buck-boost converter whose gain is two times higher than that of the conventional buck-boost converter; however, its voltage gain is still not high enough [18]. presents a semi-quadratic high voltage gain buck-boost converter with a reasonable number of storage components; however, it requires two power switches. In addition, input ripple cancellation is only accessible at a particular duty cycle and its input ripple at other duty cycles is high [19]. presents a high step-up buck-boost converter with a semi-quadratic voltage gain. The benefits of this converter include using only one switch, continuity of input current and output polarity, and low semiconductor voltage stresses. In return, it requires a remarkably high number of passive components and its output current is still not continuous. In addition, it is not suitable for high step-down applications. The continuity of output current in DC/DC converters offers a non-pulsating power to the storage batteries or DC-links [20]. A group of studies benefits from high-frequency transformers or coupled inductors to attain higher voltage gains [21]. These converters suffer from high cost and massive ferrite cores which are usually considered as low-efficient parts of DC/DC converters. Moreover, stored energy in the leakage inductance of the transformer induces high voltage spikes on semiconductors which must be considered [22,23]. Recovering leakage inductance energy requires passive clamp circuits that increase the complexity of the converter circuit. In addition to mentioned DC/DC topologies and in order to have less power loss while providing high voltage gain, modular and interleaved buck-boost DC/DC converters have been proposed recently. As the total power is shared between units, current and voltage stress is lower in switches and as a result, efficiency increases [24,25]. However, increasing the number of switching elements will increase the costs and complexity in the control system.

In this paper, a new semi-quadratic transformerless high step-up buck-boost converter is proposed which has the main following features compared to other presented topologies:

• Benefits from only one switch and 3 diodes which make an easier control and driving circuit, and consequently, minimize the complexity and cost.
• High static voltage gain.
• Continuity of Input and output currents.
• Bi-polar voltage characteristic of the derivative form of the proposed converter.

This paper is organized as follows. In Section 2, the proposed converter topology is described in detail. Furthermore, its steady-state analysis in Continuous Conduction Mode (CCM) is studied. Following that, the derivative model of the proposed converter is presented which is achieved by rearranging elements (Section 3). This version of the proposed quadratic buck-boost converter is a reasonable choice since the extremely high-duty cycle problem is noteworthy for both stepping-down and stepping-up modes. In Section 4, a detailed comparison between the proposed converter and similar types that have been proposed in the literature is made. Then in Section 5, the state space equation of the proposed converter and the bode diagram of the duty cycle to output voltage transfer function is provided. Section 6 presents the simulation results of the converter presented in Sections 2, 3 of this paper. Section 7 includes investigating the experimental results of the proposed high step-up semi-quadratic single switches buck-boost converter (proposed in Section 2), which verifies the theoretical and simulation results.

## 2 Semi-quadratic single-switch buck-boost converter

The proposed semi-quadratic buck-boost converter is introduced in Fig. 1a. The converter is originally derived from two primary boost converters and one basic buck converter. In addition, the proposed converter uses just one switch for driving, which significantly reduces the complexity of gate driving and control circuits. On the other hand, Fig. 1b shows a most recent proposed quadratic buck-boost converter which consists of two switches [26]. While two converters have continuous input and output currents, the proposed converter has a reverse output voltage.

**Fig. 1a:**
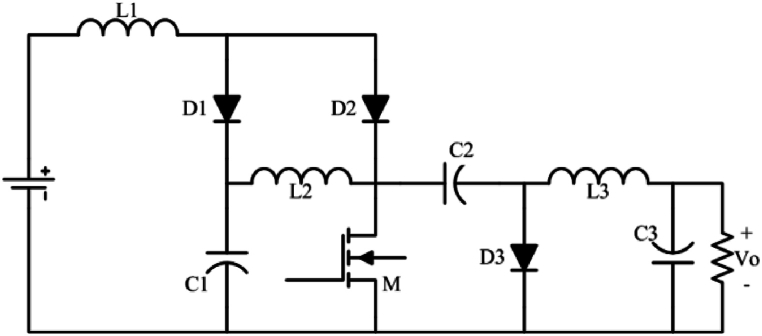
Proposed single-switch semi-quadratic buck-boost converter.

**Fig. 1b:**
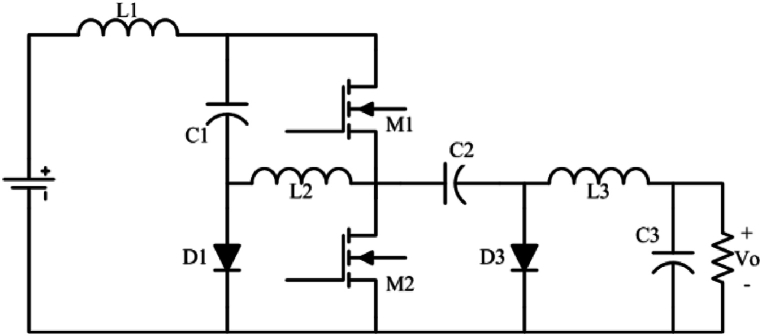
Quadratic buck-boost converter proposed in Ref. [26].

### 2.1 Steady state analysis

**Fig. 2:**
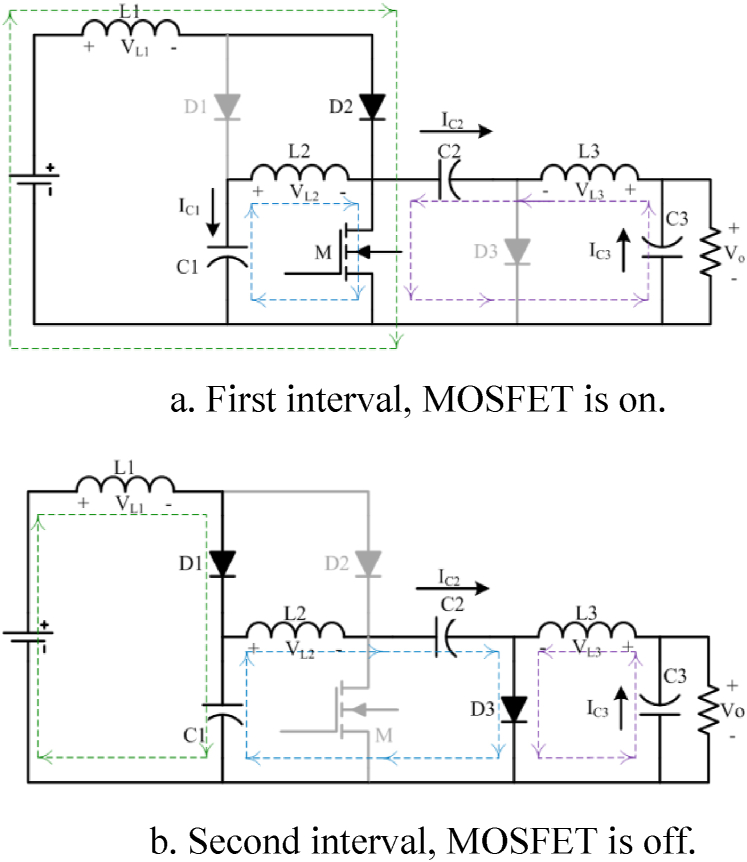
Two main operation modes of the proposed converter, assuming the CCM operation condition.

Assuming the C.C.M operating condition, there are two intervals for proposed converter which is demonstrated in Fig. 2.

• **Mode 1:**

The MOSFET (M) is switched on. In this condition, D2 is forward biased and turned on. Two other diodes (D1, D3) are reverse biased. Then, three inductors are charged by the MOSFET path. From KCL and KVL, sets of Eq. (1) can be derived:

1 $$\begin{array}{cc} \left\{ \begin{array}{c} V_{L1}=V_{in}\\ V_{L2}=V_{C1}\\ V_{L3}=V_{C2}-V_{C3} \end{array}\right. & \left\{ \begin{array}{c} I_{C1}=-I_{L2}\\ I_{C2}=-I_{L3}\\ I_{C3}=I_{L3}-I_{R} \end{array}\right. \end{array}$$

• **Mode 2:**

Stopping triggering makes the MOSFET (M) to be turned off and the continuity of the inductors’ currents makes diodes (D1, D3) to be turned on. Therefore, D2 is reverse-biased and turned off. In addition, L1, L2, L3 are discharged to C1, C2, C3, respectively.

Appling the KCL and KVL, inductors voltages(V_L1_,V_L2_,V_L3_) and capacitors currents(I_C1_,I_C2_,I_C3_) can be derived as Eq. (2):

2 $$\begin{array}{cc} \left\{ \begin{array}{c} V_{L1}=V_{in}-V_{C1}\\ V_{L2}=V_{C1}-V_{C2}\\ V_{L3}=-V_{C3} \end{array}\right. & \left\{ \begin{array}{c} I_{C1}=I_{L1}-I_{L2}\\ I_{C2}=-I_{L2}\\ I_{C3}=I_{L3}-I_{R} \end{array}\right. \end{array}$$

Moreover, the equations for capacitor voltages can be derived from charge current balance principle as set of Eq. (3):

3 $$\begin{array}{c} V_{C1}=\frac{1}{\left(1-D\right)}V_{in}\\ V_{C2}=\frac{1}{{\left(1-D\right)}^{2}}V_{in}\\ V_{o}=V_{C3}=\frac{-D}{{\left(1-D\right)}^{2}}V_{in} \end{array}$$

where, the last equation in Eq. (3) is static voltage gain of the proposed converter. Inductor currents relationships are obtained from charge current balance principle of the capacitors (Eq. (4)):

4 $$\begin{array}{l} I_{L1}=\frac{D}{{\left(1-D\right)}^{2}}\frac{V_{O}}{R}\\ I_{L2}=\frac{D}{\left(1-D\right)}\frac{V_{O}}{R}\\ I_{L3}=\frac{V_{O}}{R} \end{array}$$

It is apparent that this converter works as a high step-up buck-boost converter with a wide output voltage range. As a result, there is a very good control over the level of converter output voltage. Also, the current of the source is equal to the first inductor current and it is continuous. Fig. 5 shows the curve of the voltage gain of the proposed converter versus the duty cycle.

**Fig. 3:**
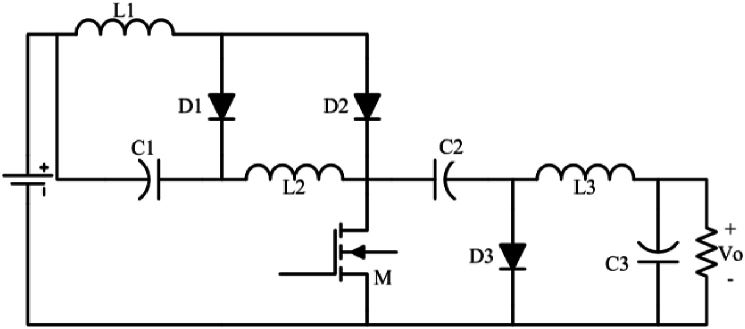
The proposed high step-up buck-boost converter schematic.

In Fig. 3, another high step-up buck-boost converter is introduced which has lower stress voltage on C_1_, in comparison with converter introduced in Fig. 1a while the proposed converter (Fig. 3) uses the same component number to the converter illustrated in Fig. 1a, its first capacitor stress is D times, i.e. $V_{C1}=\frac{D}{\left(1-D\right)}V_{in}$ in the converter of Fig. 3. Note that the operation principle and the output gain voltage of the two converters (Fig. 1a and Fig. 3) are the same, exactly.

### 2.2 Current and voltage stress of switching elements

The current and voltage stress of switching elements are one of the important issues in designing the power converters. This is evaluated in the following section.

#### 2.2.1 Current stress of switch and diodes

In this section, the current stresses of switches are discussed. With assuming high inductance value for inductors, inductor current ripples can be ignored. Eq. (5) represents current stress of the MOSFET:

5 $$I_{M}=D\left(I_{L1}+I_{L2}+I_{L3}\right)=\frac{D}{{\left(1-D\right)}^{4}}\frac{V_{in}}{R}$$

Also, Eq. (6) denotes the current stress of D2.

6 $$I_{D2}=\left(D\right)I_{L1}=\frac{D^{3}}{{\left(1-D\right)}^{4}}\frac{V_{in}}{R}$$

#### 2.2.2 Voltage stress of switch and diodes

The voltage stress across the switch can be derived as Eq. (7):

7 $$V_{M}=V_{C2}=\frac{1}{{\left(1-D\right)}^{2}}V_{in}$$

Also, the voltage stress values of diodes can be obtained from Eq. (8):

8 $$\begin{array}{l} V_{D1}=V_{C1}=\frac{1}{\left(1-D\right)}V_{in}\\ V_{D2}=V_{C2}-V_{C1}=\frac{D}{{\left(1-D\right)}^{2}}V_{in}\\ V_{D3}=V_{C2}=\frac{1}{{\left(1-D\right)}^{2}}V_{in} \end{array}$$

### 2.3 Current ripple of inductors and voltage ripple of capacitors

Current ripple of inductors and voltage ripple of capacitors are summarized in Table 1.

**Table 1:** Current ripple of inductors and voltage ripple of capacitors.

Current ripple of inductors	$\Delta I_{L1}=\frac{V_{in}D}{L_{1}f_{S}}$	$\Delta I_{L2}=\frac{V_{in}D}{{\left(1-D\right)}^{2}f_{S}L_{2}}$	$\Delta I_{L3}=\frac{V_{in}}{\left(1-D\right)f_{S}L_{3}}$
Voltage ripple of capacitors	$\Delta V_{C1}=\frac{D^{2}V_{O}}{\left(1-D\right)RC_{1}f_{S}}$	$\Delta V_{C2}=\frac{DV_{O}}{RC_{1}f_{S}}$	$\Delta V_{C3}=\frac{\left(1-D\right)V_{O}}{8DC_{1}L_{3}{f_{S}}^{2}}$

In addition, Eq. (9) determines the restrictions of the converter for operating in continuous conduction mode:

9 $$\begin{array}{c} I_{L1}\geq \Delta I_{L1}\\ I_{L2}\geq \Delta I_{L2}\\ I_{L3}\geq \Delta I_{L3} \end{array}\to \begin{array}{c} L_{1}\geq \frac{R{\left(1-D\right)}^{3}}{Df_{s}}\\ L_{2}\geq \frac{R}{Df_{s}}\\ L_{3}\geq \frac{R}{Df_{s}} \end{array}$$

Considering all of the above restrictions for designing the converter will guarantee the C.C.M operation of the converter.

## 3 A quadratic single-switch buck-boost converter with two outputs

**Fig. 4:**
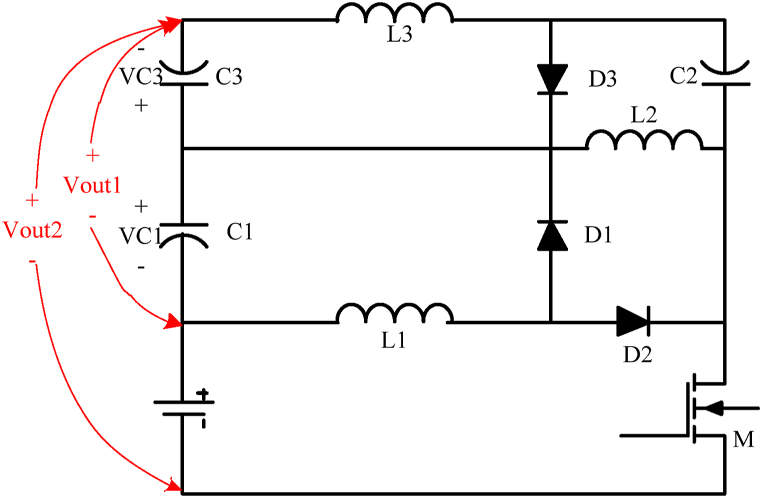
Quadratic single switch buck-boost converter.

Fig. 4 illustrates a quadratic buck-boost converter. This converter is a derivative form of topology introduced in Fig. 1a which needs a single switch and three diodes. Its steady-state analysis is the same as the converter introduced in Fig. 1a. Likewise the converter introduced in Fig. 1a, Fig. 3, this converter has two intervals. In the first interval, MOSFET and D2 are on (D1 and D3 are off), and in the second interval D1, and D3 are on.

**Fig. 5:**
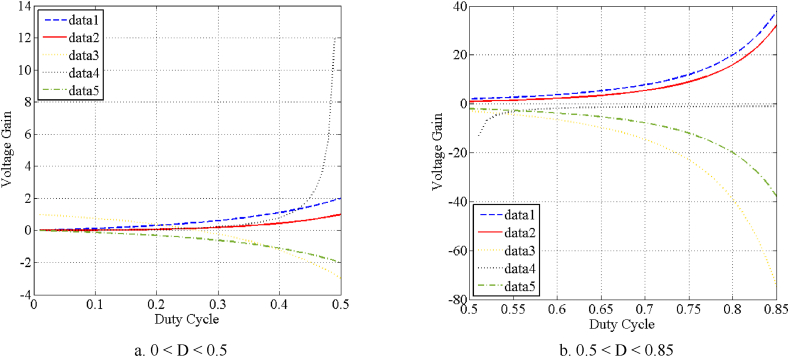
Voltage gain comparison of relevant converters. Data1: [27,32]. Data 2: [9,26,28]. Data 3: proposed converter (V_out2_, Fig. 4). Data 4: [29]. Data 5: proposed converter (Fig. 1a).

Eq. (10) can be derived that shows the capacitors voltages relations of the quadratic buck-boost converter:

10 $$\begin{array}{c} V_{C1}=\frac{D}{\left(1-D\right)}V_{in}\\ V_{C2}=\frac{D}{{\left(1-D\right)}^{2}}V_{in}\\ V_{C3}=\frac{D}{{\left(1-D\right)}^{2}}V_{in} \end{array}$$

Then, static voltage gain of both outputs can be found as follows:

11 $$V_{out1}=V_{C1}-V_{C3}=\frac{D}{\left(1-D\right)}V_{in}-\frac{D}{{\left(1-D\right)}^{2}}V_{in}=\frac{-D^{2}}{{\left(1-D\right)}^{2}}V_{in}$$

$$V_{out2}=V_{in}+V_{C1}-V_{C3}=V_{in}+\frac{D}{\left(1-D\right)}V_{in}-\frac{D}{{\left(1-D\right)}^{2}}V_{in}=\frac{1-2D}{{\left(1-D\right)}^{2}}V_{in}$$

As can be figured out, two outputs with different voltage gains can be derived from the proposed converter. Both voltage gains of the proposed converter versus the duty cycle are depicted in Fig. 5.

## 4 Comparison of the proposed converter with similar types

Table 2 conducts a comparison between the proposed converter and some related converters in different aspects such as the number of components, the voltage gains, and voltage stress of switching components. As can be observed, the proposed topology and its derivative in this paper compared to other similar topologies is benefiting from only one switch and three diodes. The additional benefit is that this makes the driving and control circuit of the converter much easier. Furthermore, as the ground of the switch is connected to the input voltage source, the driver circuit becomes simpler without the need for an additional isolated power supply. Furthermore, according to Eq. (11), the second output voltage is bipolar. The voltage gain of relevant converters (mentioned in Table 2) are illustared in Fig. 5.

**Table 2:** Comparison between the proposed converter and some relevant converters.

Topology	Proposed converter	Proposed converter	[9]	[27]	[26]	[28]	[29]	[30]	[31]	SIBB2C
	(Fig. 1aAnd Fig. 3)	(Fig. 4)								[32]
switches	1	1	1	2	2	2	2	2	2	1
diodes	3	3	5	2	2	2	2	2	2	3
inductors	3	3	3	2	3	2	2	2	3	2
capacitors	3	3	3	2	3	2	2	2	3	2
voltage gain	$\frac{-D}{{\left(1-D\right)}^{2}}$	$\begin{array}{l} Out1:\frac{D^{2}}{{\left(1-D\right)}^{2}}\\ Out2:\frac{1-2D}{{\left(1-D\right)}^{2}} \end{array}$	$\frac{D^{2}}{{\left(1-D\right)}^{2}}$	$\frac{D}{{\left(1-D\right)}^{2}}$	$\frac{D^{2}}{{\left(1-D\right)}^{2}}$	$\frac{D^{2}}{{\left(1-D\right)}^{2}}$	$\frac{D^{2}}{\left(1-2D\right)}$	$\frac{D}{{\left(1-D\right)}^{2}}$	$\frac{D}{{\left(1-D\right)}^{2}}$	$\frac{D}{{\left(1-D\right)}^{2}}$
voltage stress of the switches	$\frac{V_{o}}{D}$	$\frac{V_{out1}}{D^{2}}$	$\frac{V_{o}}{D^{2}}$	$\frac{\left(1-D\right)V_{o}}{D}$	$\frac{V_{o}}{D}$	$\frac{\left(1-D\right)V_{o}}{D^{2}}$	$\frac{\left(1-D\right)V_{o}}{D^{2}}$	$\frac{\left(1-D\right)V_{o}}{D}$	$\frac{\left(1-D\right)V_{o}}{D}$	$\frac{V_{o}}{D}$
				$V_{o}$	$\frac{V_{o}}{D^{2}}$	$\frac{V_{o}}{D}$	$\frac{V_{o}}{D}$	$\frac{V_{o}}{D}$	$\frac{V_{o}}{D}$	
voltage stress of the diodes	$\frac{\left(1-D\right)V_{o}}{D}$	$\frac{\left(1-D\right)V_{out1}}{D}$	$\frac{\left(1-D\right)V_{o}}{D^{2}}$ $\frac{V_{o}}{D}$	$\frac{\left(1-D\right)V_{o}}{D}$	$\frac{\left(1-D\right)V_{o}}{D}$	$\frac{\left(1-D\right)V_{o}}{D^{2}}$	$\frac{\left(1-D\right)V_{o}}{D^{2}}$	$\frac{\left(1-D\right)V_{o}}{D}$	$\frac{\left(1-D\right)V_{o}}{D}$	$V_{o}$
										$\frac{V_{o}}{D}$
	$V_{o}$	$\frac{\left(1-D\right)V_{out1}}{D}$	$\frac{V_{o}}{D^{2}}$							
				$\frac{V_{o}}{D}$	$\frac{V_{o}}{D}$	$\frac{V_{o}}{D}$	$\frac{V_{o}}{D}$	$\frac{V_{o}}{D}$	$\frac{V_{o}}{D}$	
	$\frac{\left(1-D\right)V_{o}}{D}$	$\frac{V_{out1}}{D^{2}}$	$\frac{\left(1-D\right)V_{o}}{D^{2}}$ $\frac{V_{o}}{D^{2}}$							$\frac{\left(1-D\right)V_{o}}{D}$
Output polarity	negative	positive/negative	positive	positive	positive	positive	positive	positive	negative	negative
Continuity of input current	Yes	yes	yes	yes	yes	no	no	Yes	yes	no
Continuity of output current	Yes	yes	yes	no	yes	no	yes	No	yes	no
Maximum Reported Efficiency (Experimental)	89.5 %@		90 % @	95.2 % @	90.9 %@	90 %@	Not mentioned	93 %@	90.5 % @	92 %@
	V_in_ = 24 V		V_in_ = 30 V	V_in_ = 40 V	V_in_ = 12 V,	V_in_ = 18 V		V_in_ = 18 V	V_in_ = 18 V	V_in_ = 24 V
	V_out_ = 48 VI_load_ = 1 A		V_out_ = 65 VI_load_ = 1.08 A	V_out_ = 120 VI_load_ = 0.4 A	V_out_ = 24 VI_load_ = 0.5 A	V_out_ = 40.5 VI_load_ = 0.27 A		V_out_ = 48 VI_load_ = 0.4 A	V_out_ = 48 VI_load_ = 0.4A	V_out_ = 48 VI_load_ = 0.08 A
	F_s_ = 50 kHz		F_s_ = 40 kHz	F_s_ = 20 kHz	F_s_ = 60 kHz	F_s_ = 20 kHz		F_s_ = 40 kHz	F_s_ = 40 kHz	Fs = 25 kHz

## 5 Small signal modeling of the converter

In order to analyze the dynamic of the converter, small-signal modeling of the proposed converter is studied in this section. This can be done by linearizing the state space equations of the converter with an average modeling approach. Regarding Eq. (1) and Eq. (2), differential equations of the converter can be written as follows:

12 $$\begin{array}{l} L_{1}\frac{dI_{L_{1}}}{dt}=V_{in}-D^{\prime }V_{C_{1}}\\ L_{2}\frac{dI_{L_{2}}}{dt}=V_{C_{1}}-D^{\prime }V_{C_{2}}\\ L_{3}\frac{dI_{L_{3}}}{dt}=DV_{C_{2}}-V_{C_{3}}\\ C_{1}\frac{dV_{C_{1}}}{dt}=DI_{L_{1}}-I_{L_{2}}\\ C_{2}\frac{dV_{C_{2}}}{dt}=-DI_{L_{3}}-D^{\prime }I_{L_{2}}\\ C_{3}\frac{dV_{C_{3}}}{dt}=I_{L_{3}}-\frac{V_{C_{3}}}{R_{L}} \end{array}$$

In average modeling, each state parameter is modeled with a DC term plus its perturbation value near operation point as below:

13 $$F\left(t\right)=F_{DC}+{\hat{f}}_{AC}$$

hence, by substituting Eq. (13) in Eq. (12) and doing some modification, state space equation of the converter is found as Eq. (14):

14 $$\left[\begin{array}{c} \frac{d{\hat{i}}_{L_{1}}}{dt}\\ \frac{d{\hat{i}}_{L_{2}}}{dt}\\ \frac{d{\hat{i}}_{L_{3}}}{dt}\\ \frac{d{\hat{v}}_{C_{1}}}{dt}\\ \frac{d{\hat{v}}_{C_{2}}}{dt}\\ \frac{d{\hat{v}}_{C_{3}}}{dt} \end{array}\right]=\left[\begin{array}{cccccc} 0 & 0 & 0 & -\frac{D^{\prime }}{L_{1}} & 0 & 0\\ 0 & 0 & 0 & \frac{1}{L_{2}} & -\frac{D^{\prime }}{L_{2}} & 0\\ 0 & 0 & 0 & 0 & \frac{D}{L_{3}} & -\frac{1}{L_{3}}\\ \frac{D}{C_{1}} & -\frac{1}{C_{1}} & 0 & 0 & 0 & 0\\ 0 & -\frac{D^{\prime }}{C_{2}} & -\frac{D}{C_{2}} & 0 & 0 & 0\\ 0 & 0 & \frac{1}{C_{3}} & 0 & 0 & -\frac{1}{R_{L}C_{3}} \end{array}\right]\left[\begin{array}{c} {\hat{i}}_{L_{1}}\\ {\hat{i}}_{L_{2}}\\ {\hat{i}}_{L_{3}}\\ {\hat{v}}_{C_{1}}\\ {\hat{v}}_{C_{2}}\\ {\hat{v}}_{C_{3}} \end{array}\right]+\left[\begin{array}{c} \frac{V_{C_{1}}}{L_{1}}\\ \frac{V_{C_{2}}}{L_{2}}\\ \frac{V_{C_{2}}}{L_{3}}\\ \frac{I_{L_{1}}}{L_{1}}\\ \frac{I_{L_{2}}-I_{L_{3}}}{L_{2}}\\ 0 \end{array}\right]\hat{d}$$

**Fig. 6:**
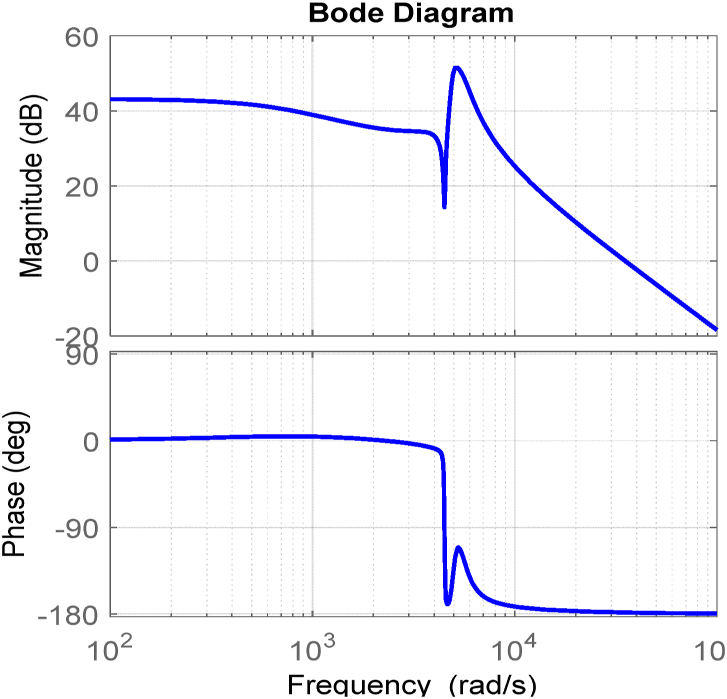
Bode diagram of duty cycle to output voltage of the proposed converter.

By using parameters of Table 3 (in section 5) and considering D = 50 %, Bode diagram of the $G_{\hat{V}\hat{d}}$ transfer function is plotted in Fig. 6.

**Table 3:** Designed parameters for simulation of the proposed buck-boost converter.

parameter	value
$V_{in}$	24
$L_{1}$	500 μH
$L_{2}$	450 μH
$L_{3}$	400 μH
$C_{1}$	30 μF
$C_{2}$	70 μF
$C_{3}$	100 μF

It can be seen that the phase margin is positive and equal to 1.66 but it is not enough to guarantee stability. As a result, a PI controller can be implemented to increase phase margin and bandwidth to satisfy the transient response of the converter.

## 6 Simulation results

In the following, the proposed high step-up semi-quadratic buck-boost and also the quadratic buck-boost converter are simulated by PSIM software.

### 6.1 Proposed high step-up buck-boost converter

In the first step, the converter presented in Fig. 1a is simulated and investigated for a 24V input. In order to ensure that the converter works in C.C.M, Eq. (9) relations are taken into account for designing the passive elements. Table 3 reveals the results of designed parameters for the simulation of the proposed buck-boost converter (considering output current = 1A, Step-up mode output voltage: $V_{o}$ = 48, R = 48, and Step-down mode output voltage: $V_{o}$ = 15, R = 15). In both modes switching frequency has been considered 50 kHz and the duty cycle was chosen based on Eq. (3).

**Fig. 7:**
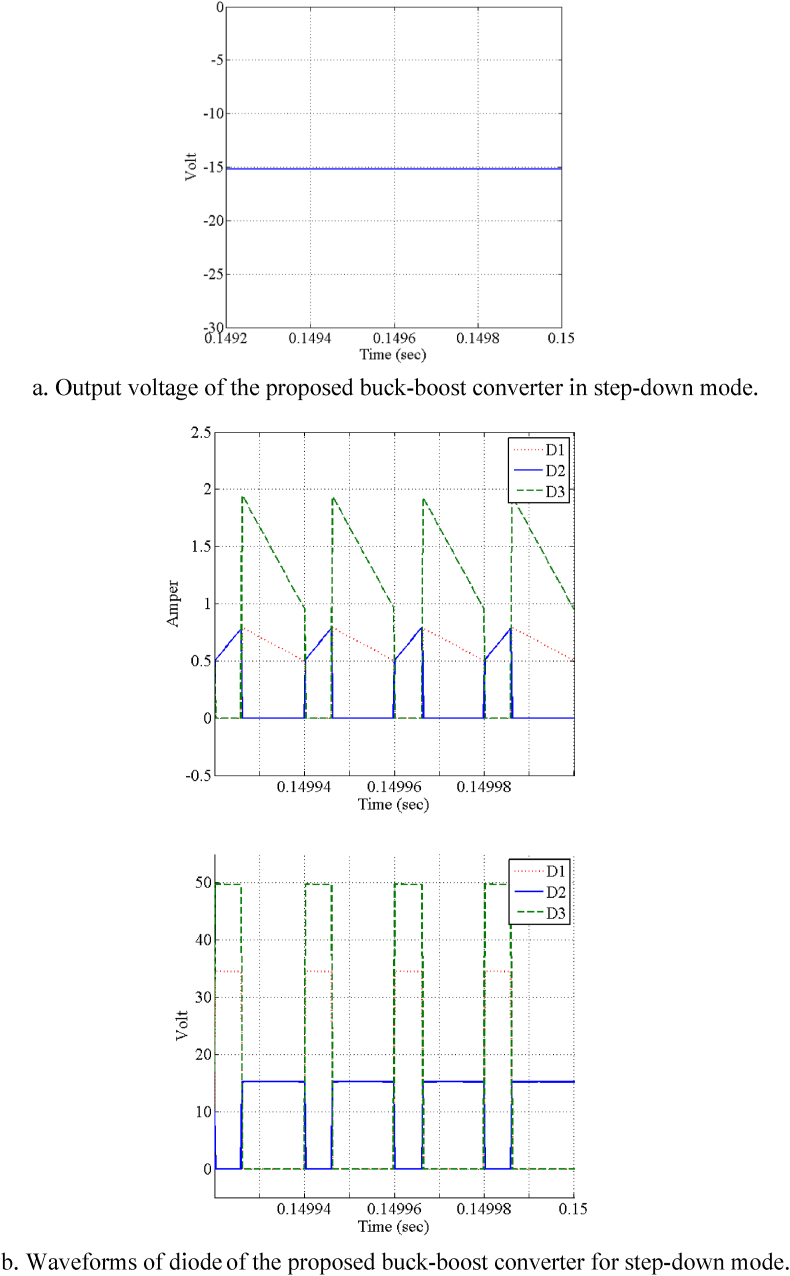
Simulation results of the proposed high step-up/step-down buck boost converter in step-down mode ($V_{in}=24V$, $V_{out}=15V$, $I_{out}=1A$).

**Fig. 8:**
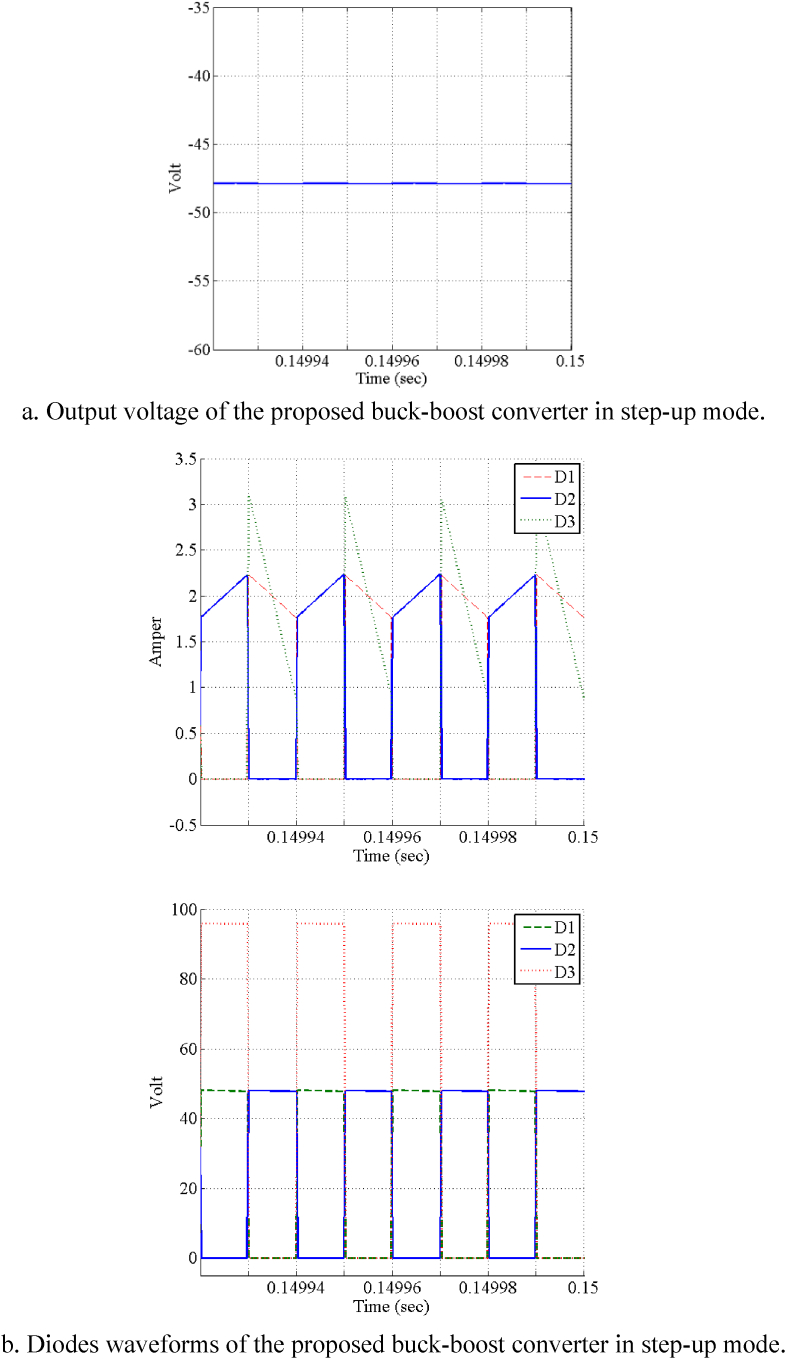
Simulation results of the proposed high step-up/step-down buck-boost converter in step-up mode ($V_{in}=24V$, $V_{out}=48V$, $I_{out}=1A$).

Fig. 7a and b show the output voltage of the proposed buck-boost converter in step-down mode and waveforms of diode of the proposed buck-boost converter for step-down mode, respectively. In addition, Fig. 8 demonstrate the simulation results of the proposed converter in step-down and step-up mode. In this figure, Fig. 8a and b show the output voltage of the proposed buck-boost converter in step-up mode and the diodes waveforms of the proposed buck-boost converter in step-up mode. It is obvious that the converter could work in C.C.M for both step-down and step-up modes. It is noteworthy that if converter does not work in C.C.M, the output voltage cannot adopt with the expected value and it is lower.

### 6.2 Quadratic buck-boost converter

**Fig. 9:**
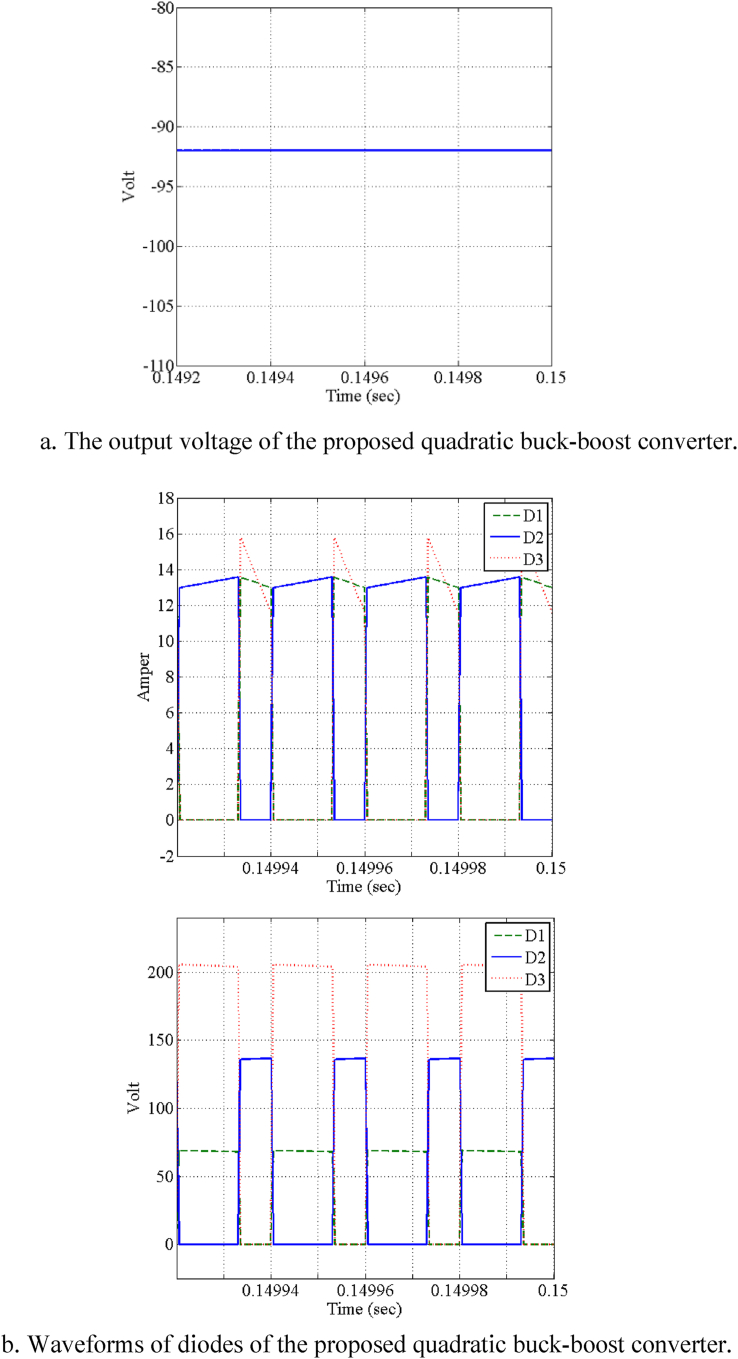
Simulation results of the proposed quadratic buck-boost converter ($V_{in}=24V$, $V_{out}=96V$, $I_{out}=4.8A$).

Assuming the parameters of Table 3, the quadratic buck-boost converter (Fig. 4) is also simulated using the PSIM software ($V_{out}=96V$, $I_{out}=4.8A$). The results are illustrated in Fig. 9 (Fig. 9a shows the output voltage of the proposed quadratic buck-boost converter - Fig. 9b shows the waveforms of diodes of the proposed quadratic buck-boost converter). As it can be understood, the converter has an appropriate performance.

## 7 Experimental results

### 7.1 Converter operation

**Fig. 10:**
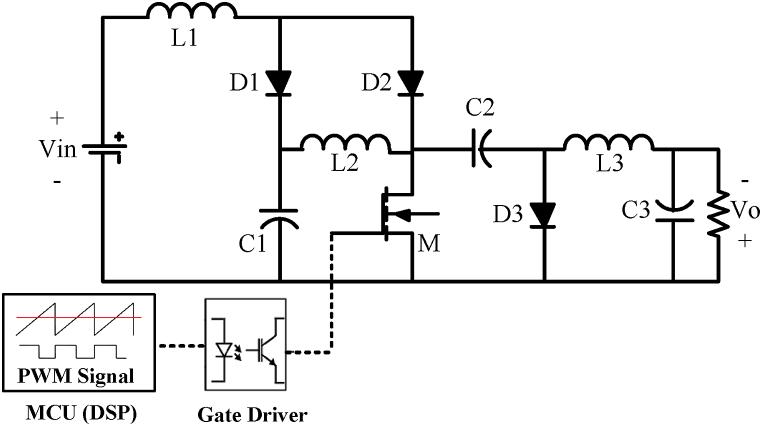
Power and control circuit of proposed converter used for obtaining experimental results.

In order to validate theoretical and simulation results in previous sections, a 50 W prototype of proposed converter (Fig. 1a) has been built. The schematic that shows the connection between power and driving signal is depicted in Fig. 10. The driving circuit consists of a DSP for generating PWM signal and a gate driver to drive MOSFET switch. Regarding the power circuit, designed parameters that were used to obtain experimental results are summarized in Table 4.

**Table 4:** Summary of design parameters for proposed converter.

Parameter	Value
Nominal Power	50 W
Switching Frequency	50 kHz
Inductors L1, L2 and L3	
Inductance Value	500 μH/450 μH/400 μH
Core types	Ferrite type: EE 40/EE 33/EE 33
Winding	78 turn, AWG17/61 turn, AWG19/61 turn, AWG19
Air gap	2.1 mm/1.28mm/1.45 mm
R_L_	25 mΩ/22.5 mΩ/20 mΩ
Capacitors C1, C2 and C3	
Type and Value	Electrolyte, 30 μF, 70 μF, 100 μF
MOSFET Switch (M)	
Part Number	IRFB4227
Diodes	
Part Number	MUR840

**Fig. 11:**
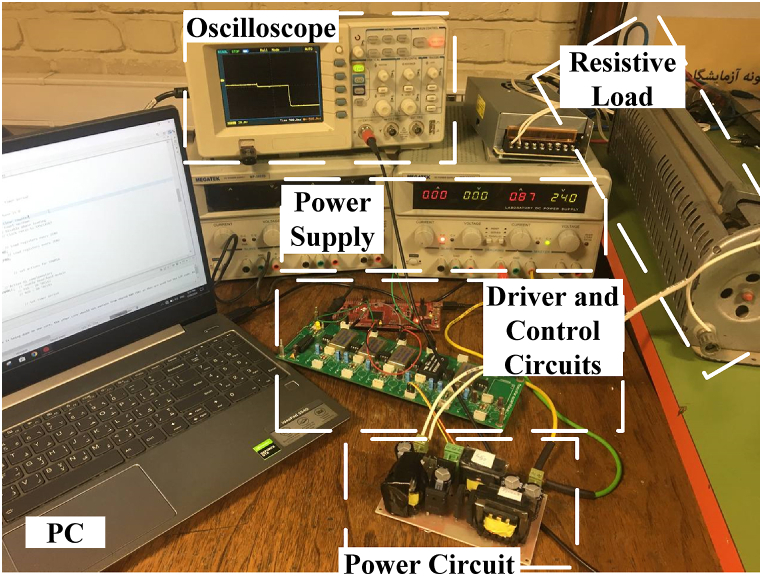
A laboratory prototype of the proposed converter.

Fig. 11 demonstrates the laboratory setup used to obtain experimental results. Experiments have been carried out for both step-down (input voltage = 24 V, output voltage = 12V) and step-up (input voltage = 24V, output voltage = 48V under full load condition) modes.

**Fig. 12:**
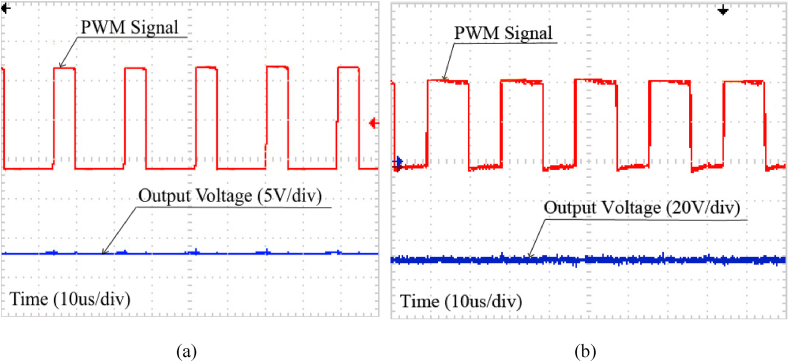
Output voltage and PWM signal of switch M in (a) Step-down mode, (b) Step-up mode.

In Fig. 12, Output voltage and PWM pulse on switch M are shown in both modes when the input voltage is 24VDC. Fig. 12a shows the output voltage for step-down mode is fixed to 12VDC and also Fig. 12b shows the output voltages for step-up is fixed to 48VDC). As is clear from this figure, the output voltages in step-down mode and step-up mode are matched due to the theoretical (duty cycle = 0.27 for step-down mode/duty cycle = 0.5 for step-up) and simulation results.

**Fig. 13:**
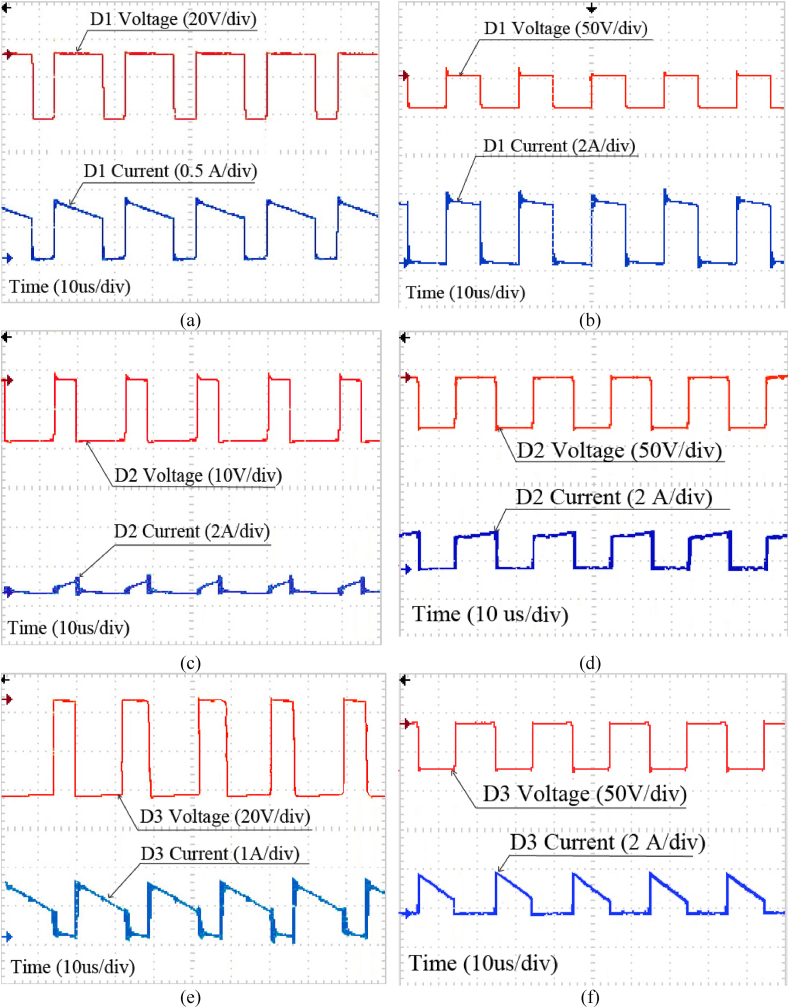
(a) Voltage and Current of D_1_ in (a) Step-down mode, (b) Step-up mode, Voltage and Current of D_2_ in (c) Step-down mode, (d) Step-up mode, Voltage and Current of D_3_ in (e) Step-down mode, (f) Step-up mode.

Also, Fig. 13(a)–(f) illustrate voltage and currents of diodes D_1_ to D_3_ in both modes.

**Fig. 14:**
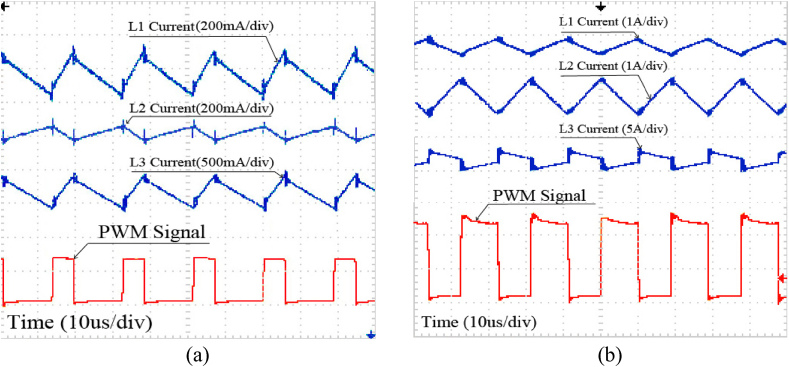
Currents of L_1_, L_2_ and L_3_ in (a) Step-down mode, (b) Step-up mode.

In addition, Currents of the inductors L_1_ to L_3_ are illustrated in Fig. 14a and Fig. 14b for step-down and step-up modes, respectively.

**Fig. 15:**
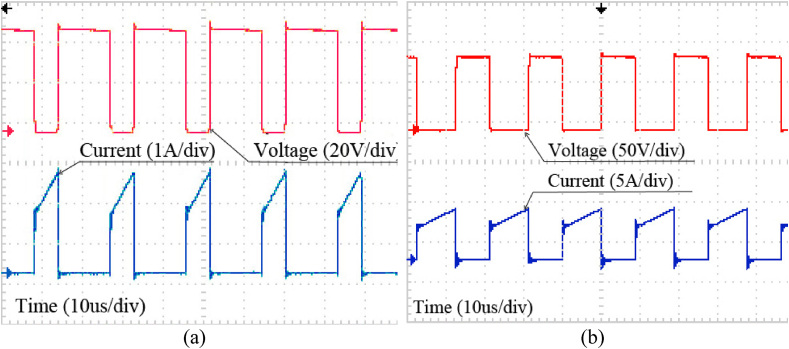
Current and Voltage of switch M in (a) Step-down mode, (b) Step-up mode.

In Fig. 15a and Fig. 15b, voltage and current across the switch M are shown in both step-down and step-up modes. As it is apparent, in step-down mode, the voltage stress of the switch is lower than in step-up mode.

**Fig. 16:**
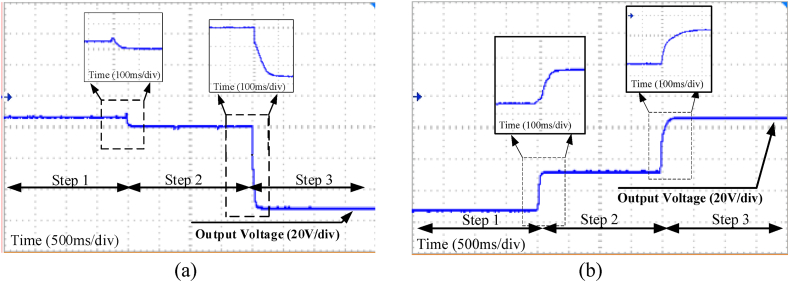
Output voltage of the converter during step change in duty cycle and input voltage (a) step-down to step-up, (b) step-up to step-down.

As a final evaluation, the operation of the converter has been examined by adding step changes in the input voltage and duty cycle. Fig. 16a shows the results of the first test scenario. In this test, during the change from steps 1 and 2, the input voltage has increased from 24 V to 36 V in a 30 % constant duty cycle. Following that, from steps 2 to 3, the duty cycle has increased to 50 % in 36V fixed input voltage. In the second test (Fig. 16b), the previous test was done oppositely. As can be seen from the results, the converter is able to operate by switching between different step-up and step-down modes while working in a wide input voltage range.

### 7.2 Efficiency

**Fig. 17:**
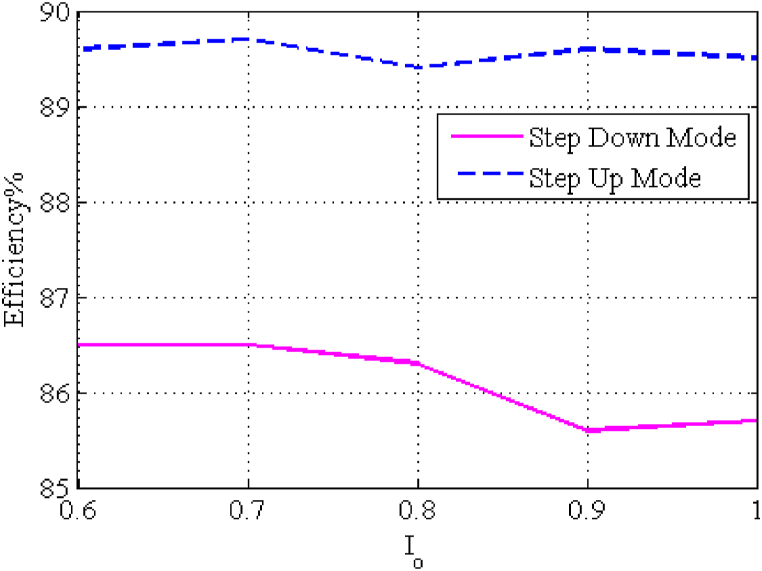
The curve of efficiency versus the output current obtained from experimental results.

**Fig. 18:**
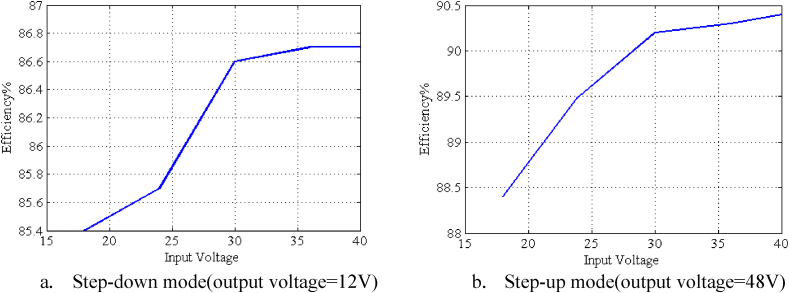
Curve of efficiency versus the input voltage when output current is constant = 1A

Fig. 17 illustrates the curve of efficiency versus the output current. As it is apparent, this converter has a higher efficiency range in step-up mode rather than step-down mode. Fig. 18a and b also show the curves of efficiency in two step-up/down modes while considering the output current constant and changing input voltage value.

## 8 Conclusion

In this paper, three single-switch buck-boost converters were proposed. The input and output side currents of all three proposed converters are continuous. The first (Fig. 1a) and second (Fig. 3) converters are high step-up buck-boost converters, while the last one (Fig. 4) is a quadratic buck-boost converter which is appropriate for high step-up/step-down applications. These converters also have the benefit of using only one switching element which makes the control and driving circuit easier than similar topologies. Following that, continuous conduction mode analyses of proposed converters and equations regarding the design of power elements were presented. The theoretical results were verified by simulation using PSIM software. Moreover, a 50 W laboratory prototype of the step-up converter was implemented and the experimental results were evaluated. The experimental results show that the converter has a higher efficiency (maximum 89.5) range in step-up mode, rather than the step-down mode (maximum 86.5) by varying the load current. This issue could be argumened of the gain of implemented converter.

## Data availability statement

No data was used for the research described in the article.

## Funding statement

The authors did not receive support from any organization for the submitted work.

## CRediT authorship contribution statement

**Mohammad Lotfi-nejad:** Project administration, Formal analysis, Data curation, Conceptualization. **Hossein Hajisadeghian:** Writing – review & editing, Writing – original draft, Resources, Methodology, Investigation, Funding acquisition. **Amir Abbas Aghajani:** Writing – review & editing, Visualization, Investigation. **Ali A. Motie Birjandi:** Writing – original draft, Visualization, Project administration. **Ehsan Adib:** Writing – review & editing, Writing – original draft, Visualization, Validation, Supervision.

## Declaration of competing interest

This paper introduces three new topologies of non-isolated buck-boost converters with continuous input current, continuous output current and also a wide conversion range.
